# Common misunderstandings of evidence-based medicine

**DOI:** 10.1007/s00399-023-00957-0

**Published:** 2023-08-07

**Authors:** Frank Semrau, Pamela Aidelsburger, Carsten Walter Israel

**Affiliations:** 1 Consultant in Health Care, Dr.-August-Wolfstieg-Str. 6, 38304 Wolfenbüttel, Germany; 2 ZOLL CMS GmbH, Emil-Hoffmann-Str. 13, 50996 Köln, Germany; 3 CAREM GmbH, Dorfstr. 32, 82549 Königsdorf, Germany; 4 Department of Cardiology, Evangelisches Klinikum Bethel, EvKB, Burgsteig 13, 33617 Bielefeld, Germany

**Keywords:** Randomized controlled trial, Intention-to-treat analysis, Per-protocol analysis, Defibrillator, Health technology assessment, Randomisierte kontrollierte Studie, Intention-to-Treat-Analyse, Per-Protokoll-Analyse, Defibrillator, „Health technology assessment“

## Abstract

Currently, most evidence assessments in guidelines or health technology assessments (HTAs) rely on the assumption that a randomized controlled trial (RCT) is always the best source of evidence. However, if the outcome in a control group is certain, e.g. death within a short time with an almost 100% chance, or if an event can only occur in the treatment group, there is no need for a randomized control group; the evidence cannot be improved by a control group, nor by an RCT design. If a cause–effect relationship is certain (“primary or direct evidence”), a therapeutic effect can be diluted in the population of an RCT by cross-over, etc. This can lead to serious misinterpretations of the effect. While experts such as the GRADE group or Cochrane institutes recommend using all available evidence, the leading approach in many guidelines and HTAs is assessing “the best available trials”, i.e. RCTs. But since RCTs only deliver probabilities of cause–effect relationships, it is not appropriate to demand RCTs for certain effects. A control group can only diminish the net value of a treatment since the outcome in the control group is subtracted from the outcome in the treatment group. Therefore, under identical circumstances, an RCT will always show lower effect rates compared to a single arm study of the same quality, for desired as well as for adverse effects. Considering these inconsistencies in evidence-based medicine interpretation, the evidence pyramid with RCTs at the top is not always a reliable indicator for the best quality of evidence.

## Abstract (de)

Aktuell verlassen sich die meisten Evidenzanalysen in Leitlinien und „health technology assessments“ (HTA) auf die Annahme, dass eine randomisierte kontrollierte Studie (RCT) immer die beste Evidenzquelle darstellt. Wenn aber das Ergebnis in einer Kontrollgruppe sicher ist, z. B. Tod innerhalb kurzer Zeit mit fast 100 %iger Sicherheit, oder wenn ein Ereignis nur in der Behandlungsgruppe auftreten kann, gibt es keinen Grund, eine Kontrollgruppe mitzuführen; die Evidenz kann dann weder durch eine Kontrollgruppe noch durch ein RCT-Studiendesign verbessert werden. Wenn eine Beziehung zwischen Wirkung und Ursache sicher ist (*primäre oder direkte Evidenz*), kann der therapeutische Effekt durch die Population in einem RCT verwässert werden durch Cross-over u. a. Dies kann zu ernsthaften Fehlinterpretationen des Effekts führen. Während Experten der GRADE-Gruppe und der Cochrane-Institute empfehlen, alle verfügbare Evidenz zu nutzen, sieht das Herangehen vieler Leitlinien und HTA die Beurteilung nur der *besten verfügbaren Studien*, d. h. der RCT, vor. Da aber RCT nur Wahrscheinlichkeiten eines Ursache-Wirkungs-Zusammenhangs nahelegen können, sind sie zur Beurteilung einiger Effekte nicht adäquat. Eine Kontrollgruppe kann den Nettonutzen einer Therapie nur vermindern, da vom Ergebnis der Behandlungsgruppe das Ergebnis der Kontrollgruppe abgezogen werden muss. Daher zeigen RCT unter identischen sonstigen Bedingungen immer einen geringeren Effekt im Vergleich zu einer prospektiven Beobachtungsstudie gleicher Qualität, dies gilt für einen Therapieeffekt ebenso wie für Nebenwirkungen. Unter Berücksichtigung dieser Widersprüche in der Interpretation der evidenzbasierten Medizin stellt die Evidenzpyramide nicht immer einen zuverlässigen Indikator für die beste Evidenzqualität dar.

## Introduction

Scientific theories and methods explaining the world or certain aspects of nature should be rational and logically consistent. Often, we accept what we learned and apply currently used proceedings without questioning them from time to time or from subject to subject. This can lead to serious mistakes. Daniel Kahneman, Nobel Prize winner and author of the book “Thinking fast and slow” would probably call this “theory induced blindness”. In this context, the authors would like to shed light on some misunderstandings of currently applied evidence-based medicine.

According to the GRADE guidelines [1], the quality of evidence is the confidence that we have in the cause–effect relationship and in the magnitude (estimates) of an effect. This is true for a body of evidence, not just for one study. In the assessment process, randomized controlled trials (RCTs) are initially considered high-quality evidence, while observational studies are considered low quality. Of note, both study types can be down- or upgraded, dependent on our confidence [2].

For most therapies and methods, the best start for gathering evidence on cause–effect relationship and effect dimensions is to pretend that we know nothing about the intervention, related effects and safety. Therefore, a study that excludes bias and potential confounders as best as possible should be the measure of choice. This is, in general, an RCT.

GRADE recommends assessing outcomes across studies and not to focus on all outcomes of one study. Furthermore, GRADE emphasizes the assessment of other factors that determine how much confidence can be placed in estimates of an effect [1]. Here it is important to stress the point that some outcomes do not need the existence of a control group in a respective trial, since the quality of those outcomes simply cannot be improved by comparative trials such as RCTs. When a parameter, such as inappropriate shocks of a defibrillator, needs to be assessed, the confidence in the detected rate of those shocks improves with the number and completeness of the observed population. A control group which shows no inappropriate shocks in a population without defibrillators does not add any confidence in the result of the treatment group.

There are few, but in fact important therapies for which efficacy and safety can be successfully determined without an RCT. Sometimes, RCTs are even obsolete, more often unnecessary [2–6]. By choosing the currently very popular approach to rely only on RCTs for every intervention and parameter, we easily miss relevant studies, misinterpret the available evidence or even turn down highly effective therapies. This leads, at best, to substantial delays in the availability of these therapies for patients.

## Methods

This manuscript is based on fundamental publications of evidence-based medicine theory from Sir Austin Bradford Hill over David L. Sacket to the GRADE group. The authors assessed the current proceedings and thoughts of today’s operationalization of evidence based medicine, revealed by study reports, medical guidelines and health technology assessments (HTAs). They then scrutinized the underlying logic and documented important but thus far often neglected logical facts as well as logical mistakes.

## Results

### RCTs

RCTs are essential tools in the armory of evidence generation. However, few RCTs are conducted perfectly and therefore, correctness of RCT results cannot be taken for granted—as is applicable for any other study type. The conditions are artificial, there may be selection bias at enrolment, some prior assumptions may turn out as wrong, and important variables may not have been taken into account. If there is a structural mistake, a wrong estimation or a service missing that is mandatory if a therapy is used in real life, statistics cannot offset this fault. Study designs are always between ideal conditions to keep the effect as large as possible (efficacy, explanatory trials) and the real life clinical situation (effectiveness, pragmatic trials).

While an RCT should answer the pivotal questions of cause–effect relationship and efficacy in the first place, today’s standard analysis type is intention-to-treat (ITT), which is blind for everything after randomization and therefore reflects rather clinical practice including potential compliance issues than the full capability of the therapy effect. Combining efficacy and effectiveness questions in one RCT may lead to rejection of a seemingly ineffective therapy due to compliance issues, while specifically addressing and fixing compliance issues would show a highly effective therapy. Furthermore, *any* crossover in a trial with an effective therapy reduces the therapy effect towards the zero hypothesis in an ITT analysis. This ramification is irrelevant if there is no therapy effect and therefore, an ITT analysis disadvantages therapies with a real effect, while it increases the chance of reaching non-inferiority even with little or no true effect.

ITT analysis and per-protocol analysis (PPA), where only (compliant) patients without protocol violations are included, answer different questions. ITT assesses the result of a therapy *assigned* to patients, PPA the result when a therapy is *applied* on patients. No analysis is better than the other, it depends on the recipient which answer is more relevant. For example, it is not of interest for a patient to know what effect a therapy offers if it is *not* appropriately applied (ITT analysis when the compliance was rather poor). Here, the PPA would be more helpful [7]. For a health system, on the other hand, it may be important to know which effect can be expected in a population in clinical practice, and here the ITT approach may make sense. ITT analysis creates a kind of buffer to prevent from overemphasizing an effect. Additionally, it strictly keeps up randomization to prevent from bias between groups. However, it depends on the specific subject, whether the strict preservation of randomization is more important than the loss of effect by including untreated patients and other study protocol violations.

It is important to recall what an RCT can tell us, and what it cannot. The results of an RCT may tell us two quite different things. On one hand, meeting the primary outcome with significance indicates there is probably a real cause–effect relationship at work. On the other hand, it points out that the magnitude of the effect in the study context is probably so large that it may seem sensible to use it on patients (instead of standard therapy or doing nothing). This is not the same. Significant results may also be found even if there is no cause–effect relationship with a therapy, and an insignificant result may be found despite a cause–effect relationship (see also “dilution effect”). A positive RCT cannot give final proof that a beneficial effect is indeed due to the therapeutic intervention. On the other hand, if a cause–effect relationship is known, a negative RCT cannot revoke this knowledge.

### Direct/primary evidence

While RCTs can only deliver probability by statistical calculations, the authors suggest introducing the term “direct/primary evidence” to the concept of evidence-based medicine: *Directly observable, instant or very timely reaction to an intervention, provable by hypothesis, test and repetition. Highest possible quality of evidence.*

RCTs are tools designed to give us probability of a cause–effect relationship. This is expressed in the 95% confidence interval or the *p*-value, telling us, e.g., that it is very likely that an intervention, e.g., a drug, caused a mortality difference seen in a trial after some years. This concept is generally accepted in the medical world, despite the fact that often we have no other, direct evidence that an intervention is effective or has a cause–effect relationship with the observed effect. In the example above, we only have a (sometimes highly probable) population effect but we cannot assign this effect to an individual patient. Neither can we tell any patient upfront that he/she will have a mortality benefit by the above mentioned drug, nor can we prove after a trial that an individual is only still alive since of the drug or died due to not taking the drug.

**Fig. 1:**
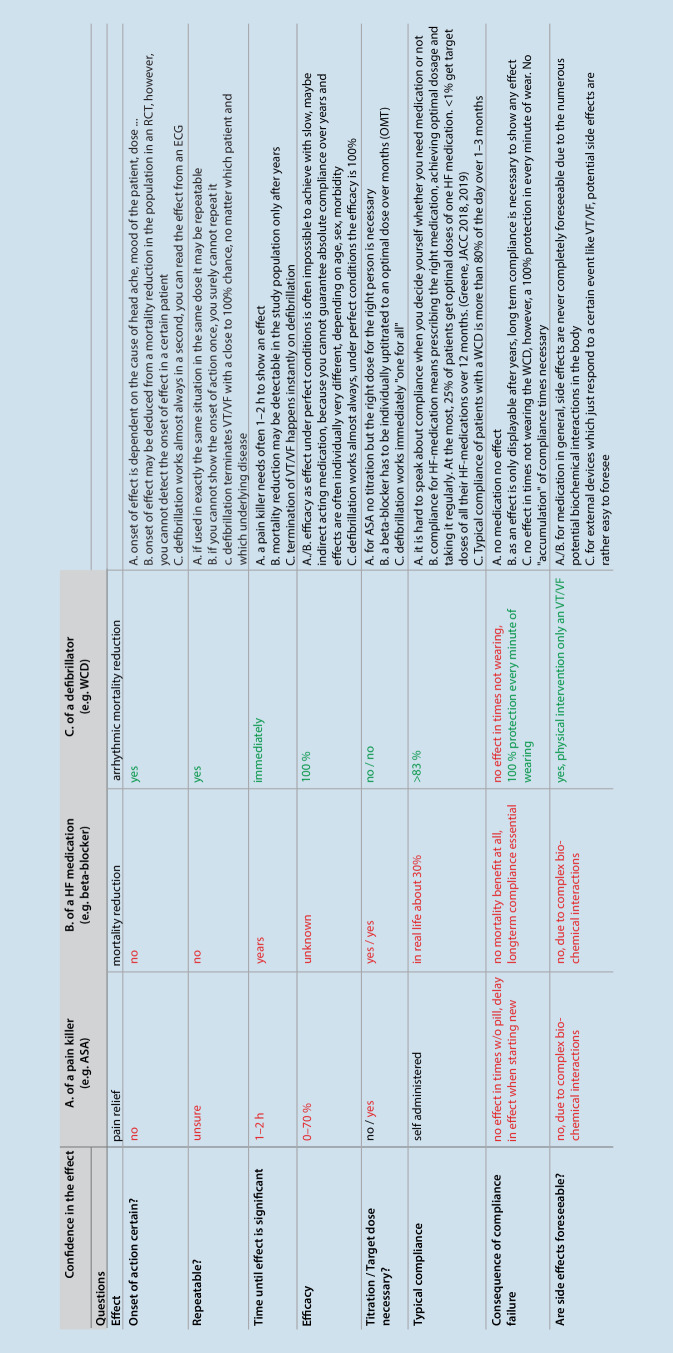
Questions on evidence for different interventions. The figure shows the answers to questions that address the general confidence in the respective effects of three different therapies. The aim is not to compare the quality of the therapies but the quality of confidence we can have in their effects. *ASA* Aspirin, *RCT* randomized controlled trial, *WCD* wearable cardioverter/defibrillator

When comparing such probable and completely accepted statistical effects to a therapy with an effect that can be directly seen and experienced, the difference is striking (Fig. 1; see also Supplement). For example, when we talk about defibrillation for ventricular fibrillation (VF), the effect can be instantly witnessed. While a patient with untreated VF dies with a chance of basically 100%, early termination of VF by defibrillation saves patients’ lives with a close to 100% chance. One can tell a given patient upfront that if VF occurs, defibrillation will terminate the otherwise lethal arrhythmia. After a trial, one can determine in every single patient whether or not the defibrillation has saved their life. Most defibrillators record the ECG during an event, thus, success or failure can be doubtlessly retraced for every single patient. The same is true for potential adverse events induced by defibrillation. Such therapies with directly perceptible effects do not need the confirmation of tools that give only probabilities of cause–effect relationships, such as RCTs. Even though the presented effect of defibrillation is clear and dramatic in magnitude, some people have doubts as long as it was not “confirmed” by an RCT—while they strongly believe in the (only very probable) effect of the above-mentioned drug that showed a statistical mortality benefit in an RCT.

### Dramatic effects

*Effect too large as to be relevantly influenced by confounding or bias. Often very timely to the intervention. Often, obvious cause*–*effect relationship.*

The GRADE guidelines 9 [2] discuss the phenomenon of dramatic effects, considering rating up quality of evidence one level when methodologically rigorous observational studies show at least a two-fold reduction or increase in risk and rating up two levels for at least a five-fold reduction or increase in risk. Therapies with a dramatic effect can escape the mantra of mandatory RCT support.

The consequences of dramatic effects of therapies with direct evidence go even further. If tested in an RCT, the RCT *must* show a significant result if the power of the study was great enough. Otherwise, the RCT is wrong. Dramatic effects are like a positive control for the effectiveness of RCTs. If RCTs were not able to show significance of dramatic effects, they were useless for medical interventions in general.

For example, defibrillators decrease arrhythmic mortality—not necessarily total mortality—by terminating lethal arrhythmic events with high efficacy. Thus, a respective RCT *must* show significant arrhythmic mortality reduction by a defibrillator, as long as the device (implantable cardioverter/defibrillator, ICD, wearable cardioverter/defibrillator, WCD, or automatic external defibrillator, AED) had the chance to detect and treat a sufficient number of events. This depends on the risk for sudden cardiac arrest (SCA) due to VF in the treated population as well as the *availability of the events for the defibrillator*. The last premise is not an issue for an ICD since the implanted device works automatically and is always active. In contrast, the WCD requires compliance since it must be worn to be able to act appropriately. An AED requires a bystander who witnesses the event, has access to an AED device, and is able and willing to use it appropriately. An RCT with any of these types of defibrillators may be “negative,” i.e. fail to show a significant arrhythmic mortality reduction, since the risk of SCD was too low in the included patient population, and/or since the WCD was not sufficiently worn, or since most SCA events were not witnessed and therefore the AED was not sufficiently used.

However, the interpretation of a potentially insignificant arrhythmic mortality reduction in an RCT would never be that defibrillators do not reduce arrhythmic mortality. A lack of significance in arrhythmic mortality reduction must be due to the RCT design, patient inclusion criteria, physician and/or patient adherence to the protocol, data collection, data interpretation, or insufficient power.

### The dilution effect of efficacy

Even when the efficacy of an intervention for the individual treated patient is close to 100% (e.g., for terminating a deterministic life-threatening event), the measured effectiveness can be considerably lower in a population where not all patients experience such an event. If only one in 100 individuals suffers from such an event, the measured effectiveness is 1% instead of 100%. This is not a fault of the therapy since the efficacy/effectiveness for a patient with an event does not change. The effectiveness (of the population) rises and falls with the risk of a population to experience such an event. Under such conditions, it makes sense to condense the risk in the treated population and to make sure the device has access to every occurring event. On the other hand, it makes no sense to conclude that the therapy does not work.

Of note, there are at least two meanings of “low risk” group. It can mean that all patients have a low risk and therefore, no one will suffer from a serious event. In the dilution example, it means there are only few patients in a group that will, however, suffer from a life-threatening event. When estimating the true mortality risk in a population independently from an assessed therapy, it is recommended to also look for mortality in the already selected patient group *before* randomization. The enrolment procedure and randomization takes some time. The fact that mortality in a selected group occurs prior to randomization does not make it unnecessary to consider, especially, whether the therapy of interest had had the potential to save some of those patients.

### How to consider evidence

If available, RCT as well as other prospective and retrospective studies should always be assessed, parameter for parameter simultaneously. As early as 1965, Sir A. Bradford Hill wrote in his famous article, The Environment and Disease: Association or Causation?, “I would myself put a good deal of weight upon similar results reached in quite different ways, e.g. prospectively and retrospectively.” Looking exclusively at RCTs represents a bias *per se*. If RCT and observational study results differ considerably, one needs to explain why, in the first place. That process should produce the most reliable studies, parameters, effects, and results (see also [8]).

Furthermore, logic uncovers that an RCT is not the best possible study design to assess evidence. Randomization is a valuable, sophisticated process to avoid confounding and bias while composing the two groups of an RCT. This way, two groups with similar base line characteristics, risk factors, and predispositions are generated, and are expected to behave similarly. However, better than *similar* are *identical* baseline characteristics. Thus, if it is possible to assess intervention and non-intervention with the same, identical group, this is far better than randomization. Despite this fact, as those studies are mostly not RCTs, they would be disregarded in many scopes of assessments, like some HTAs.

### When a control group is unnecessary

In general, a control group is necessary when a baseline for an effect is essential to estimate a net effect of an intervention. As a consequence, a control group is usually unnecessary if the outcome of a control group for a specific parameter is already quite clear. If an outcome parameter of a certain device that is unquestionably associated with the intervention, e.g., inappropriate shocks with a defibrillator, shall be measured in a trial, there is no need for a comparison group since the inappropriate shock rate in a control group without the device can only be zero. Consequently, the quality of evidence for this parameter cannot be improved by a control group, nor by an RCT. The GRADE group states, “A large series of representative patients undergoing colonoscopy will provide high-quality evidence on the risk of perforation associated with colonoscopy, when we are certain that the incidence of spontaneous colon perforation in patients not undergoing colonoscopy is very low” [2].

This is also true for effectiveness, e.g., when we are assessing an intervention with a dramatic effect on mortality. If the normal course of a disease or condition is deterministic (e.g., death in a short appraisable period of time) a control group is superfluous. Every saved or prolonged life must be caused by the intervention, in this case [6].

In these examples, a control group does not add any evidence and therefore, should not trigger a higher evidence level. Demanding RCTs for such parameters is simply not reasonable. Furthermore, it is not ethical to conduct RCTs to answer questions that can better be answered by other study designs—or that are already answered.

The authors generally suggest differentiating outcomes between parameters on one hand and effects on the other hand. The former *per se* do not need a comparator group, but can be characterized by their inherent reliability and general risk of bias. The latter consist of parameter changes between treatment and control group, potentially caused by the treatment, and often needing a control group for verification. The quality of evidence for parameters is different from the quality of evidence for effects. A perfectly conducted RCT that assesses weak parameters may not be very insightful.

### General rules when dealing with a control group

Evidence and accuracy of a measurement are not a benefit *per se*. Only the interpretation and the benefit for the patient make them valuable (see also [8]). While the effectiveness estimated from clinical trials should rather not be exaggerated in the first place, serious adverse events (SAE) should, on the contrary, be interpreted as rather frequent. Anticipating the effectiveness as rather low keeps the patient from ineffective treatments, anticipating the adverse events as rather high saves patients from unnecessary harms. Both concepts are completely different but serve the wellbeing of the patients.

The above-mentioned GRADE example of colon perforation reveals a general attribute of control groups and their impact on the effects of the respective verum group. Spontaneous colon perforations are rare, so we deduct from a single arm study that every perforation is due to the intervention. If we still added a control group, the number of perforations we assigned to the intervention would *decrease* by those found in the control group. As a general rule, a control group can only diminish an effect. This is true for an adverse as well as for a desired effect. Often, a control group reveals that the desired effect can also be found without the treatment (e.g., mediated by a placebo effect). The result is a diminished net effect in the treatment group. Here it makes sense to conduct a comparative study to protect patients from a potentially lower than anticipated effect.

The mechanism is the same for adverse events (AE). While all AEs in a single arm study are initially considered to be caused by the intervention, a comparison with a control group may uncover that part of those AEs are disease-related and the net AEs caused by the intervention are indeed less frequent. Thus, a comparative trial brings us nearer to the true rate of intervention induced AEs; however, if the AEs are already tolerable or even negligible in a large registry, it is not necessary or even reasonable to (demand to) conduct an RCT which could only show an even lower net AE occurrence. The hurdle of an RCT is immense and time-consuming, while the value for the patient cannot grow under these circumstances. Furthermore, the quality of evidence for the sake of the patient is certainly good enough in this example. Of note, when the goal is to discover rare AEs, a large population in a registry with non-restricted AE documentation and not an RCT is the most promising way. While a large population may expose very rare events, statistics cannot.

To sum up the insights, desired outcomes should never be exaggerated, so comparative studies (that subtract the base rates) deliver the more conservative results. In contrast, AEs should be viewed through a magnifier to protect patients, and here the single arm approach (without subtracting a base rate) represents the more conservative approach.

### Consequences for the evidence pyramid

Several slightly differing types of evidence pyramids can be found in the literature. However, all pyramids use a broad basement for the lowest level of evidence with case studies or even expert opinion, climb up over larger populations and comparative trials and have RCT and meta-analyses of RCTs as highest evidence level on the top. These pyramids may be appropriate for doubtful, mechanistically unexplained effects, when a control group baseline is needed to be subtracted from the potential treatment group effect. However, these pyramids are misleading, e.g., when it comes to parameters or effects that are strongly and uniquely associated with a specific intervention, such as appropriate or inappropriate shocks of a defibrillator. The highest level of evidence for those parameters comes certainly not from an RCT, but from large, well conducted single arm studies or registries. A comparator group has no impact on evidence quality, then. An evidence pyramid for such parameters would therefore look completely different to the usual ones. The authors recommend questioning traditional evidence quality schemes and pyramids and evaluating which evidence would be best for the specific parameter being assessed. The best available evidence most likely comes from different sources for different parameters and effects of the same intervention.

## Conclusions

In the current perception of evidence-based medicine, the RCT often seems to be the hammer and every question a nail. The authors present some aspects of evidence generation that are not nails and therefore do not need a hammer to be treated. For several outcomes, such as intervention-specific parameters, certain AEs and so-called dramatic effects, a control group does not add substantial, if any, benefit for evidence generation. In consequence, the quality of evidence cannot be improved by comparative trials, such as RCTs in those cases.

RCTs are not always the best available tool to generate evidence and therefore, the authors recommend using all available evidence, especially large consecutive populations, when assessing an intervention.

Control groups can only diminish the effects, seen in a verum group, including adverse effects. If the latter are already rare in large, well-conducted single arm registries, there is no need for confirmation by an RCT. Furthermore, the effectiveness in a population is not necessarily a good measure for the efficacy in an individual patient. Vice versa, a true effect on patient level cannot be questioned by RCT results.

The authors suggest the introduction of “direct/primary evidence” to the concept of evidence-based medicine, with the concept of hypothesis, test, and reliable repetition on the patient level. A series of questions may help to identify interventions that might exert direct evidence.

In summary, the authors suggest viewing the complete evidence for every parameter, effect and question specifically and, to speak with David L. Sackett, “(…) tracking down the best external evidence with which to answer our” [specific] “clinical question.”

## Appendix

### Supplement

#### Questions addressing the confidence in an intervention or effect

Some rather uncommon questions help to better understand how much or how little is known about a therapy without (or with very few) statistics. If one wants to assess an intervention, the following questions should be asked:

##### Can the effect directly be seen/experienced/measured?

As pointed out above, in many studies, the effect cannot be seen on an individual patient level. Often, an effect accumulates within several years and becomes just “measurable” in the population by statistics. However, an RCT can only give probability of an effect due to a cause–effect relationship. A painkiller may act within minutes or a few hours, leaving minimal doubt on the cause–effect relationship. Glasses that compensate a visual deficit have an immediate effect. When a brain stimulator can switch off and on and a patient starts or stops trembling, there can be no doubt of the cause–effect relationship. A repeatable, direct effect combined with a dramatic effect in a short time is the greatest possible effect with the best available evidence. This is true for, e.g., insulin for ketoacidosis, epinephrine in anaphylaxis, or defibrillation for ventricular fibrillation.

##### Is the onset of action in a patient certain?

As mentioned above, many studies show only the probability of a population effect, revealed after years by mere statistics. It cannot be predicted which individual patient will respond. Even in tumor therapy, it is usually impossible to say before the start of treatment which patient will respond and in which patient response will turn into a mortality benefit. Still, if the effect is significant in the population, no one will doubt the reality of the benefit. Thus, to be precise: for many successful and accepted therapies the onset of action in an individual patient is uncertain.

##### Is the effect reproducible?

Hypothesis, test, and repetition are the standard procedures of science. However, if it cannot be determined whether a patient benefitted from a therapy, the effect surely cannot be reproduced. In this case, the repetition must be performed by another study under the same conditions but with other individuals. This is why in guidelines, one successful RCT may lead to evidence level B rather than A. On the other hand, if an intervention induces a timely effect in a patient and this is repeatable in the same patient and/or other similar patients regularly, this is more evidence than an RCT can ever show for the above-mentioned therapy.

##### Can the effect only be determined statistically in a population (population effect)?

If yes, the evidence is not very strong. There is always a risk of error (*p*-value) and any positive effect requires confirmation that it occurred not only by chance, but as a consequence of the treatment and no other parameter. Confirmation can only come from other large RCTs. However, there always remains the possibility that another RCT will find different results.

## Declarations

For this article no studies with human participants or animals were performed by any of the authors. All studies mentioned were in accordance with the ethical standards indicated in each case.
